# M^2^S-YOLOv8: Multi-Scale and Asymmetry-Aware Ship Detection for Marine Environments

**DOI:** 10.3390/s26020502

**Published:** 2026-01-12

**Authors:** Peizheng Li, Dayong Qiao, Jianyi Mu, Linlin Qi

**Affiliations:** 1 School of Mechanical Engineering, Northwestern Polytechnical University, Xi’an 710072, China; dyqiao@nwpu.edu.cn; 2 China E-Tech(Ningbo)Maritime Electronics Research Institute Co., Ltd., Ningbo 315000, China; 18667381568@163.com; 3 College of Intelligent Systems Science and Engineering, Harbin Engineering University, Harbin 150001, China; lin.qi@hrbeu.edu.cn

**Keywords:** YOLO, ship detection, multi-scale attention mechanism, deformable convolution upsampling, small object regression

## Abstract

Ship detection serves as a core foundational task for marine environmental perception. However, in real marine scenarios, dense vessel traffic often causes severe target occlusion while multi-scale targets, asymmetric vessel geometries, and harsh conditions (e.g., haze, low illumination) further degrade image quality. These factors pose significant challenges to vision-based ship detection methods. To address these issues, we propose $M^{2}S\text{-}\mathrm{YOLOv}8$, an improved framework based on YOLOv8, which integrates three key enhancements: First, a Multi-Scale Asymmetry-aware Parallelized Patch-wise Attention (MSA-PPA) module is designed in the backbone to strengthen the perception of multi-scale and geometrically asymmetric vessel targets. Second, a Deformable Convolutional Upsampling (DCNUpsample) operator is introduced in the Neck network to enable adaptive feature fusion with high computational efficiency. Third, a Wasserstein-Distance-Based Weighted Normalized CIoU (WA-CIoU) loss function is developed to alleviate gradient imbalance in small-target regression, thereby improving localization stability. Experimental results on the Unmanned Vessel Zhoushan Perception Dataset (UZPD) and the open-source Singapore Maritime Dataset (SMD) demonstrate that $M^{2}S\text{-}\mathrm{YOLOv}8$ achieves a balanced performance between lightweight design and real-time inference, showcasing strong potential for reliable deployment on edge devices of unmanned marine platforms.

## 1. Introduction

With the advancement of global shipping intelligence [1,2,3] and the rapid development of the marine economy [4,5], unmanned surface vessels (USVs) have been widely applied in critical marine scenarios, including marine monitoring [6,7,8], port management [9,10], and maritime safety alerts [11,12]. The integration of deep learning has further advanced marine monitoring towards precision and unmanned operation [13], making object detection a core link in USV autonomous navigation and environmental perception—its accuracy and real-time performance directly determine the safety and reliability of the entire USV system.

However, practical marine environments pose significant challenges to object detection tasks: dense ship navigation easily leads to target occlusion; asymmetric geometric differences exist between slender targets (e.g., cargo ships, oil tankers) and short rectangular targets (e.g., tugboats, patrol boats); and complex weather conditions (such as haze and rainfall) and unstable lighting further degrade detection robustness. Additionally, the limited computational power of USV edge devices restricts the deployment of complex detection models with high computational demands, making the collaborative optimization of “accuracy and real-time performance” a key issue to address in sea surface target detection. Existing detection algorithms still have shortcomings in adapting to complex marine scenarios, failing to comprehensively solve the problems of multi-scale perception, adaptive feature fusion, and small target regression stability.

Early sea surface target detection approaches relied on manual feature engineering and statistical learning, which were limited by rigid designs unable to adapt to multi-source interference in marine environments [14,15,16,17]. For instance, Xu et al. [14] proposed an improved invariant generalized Hough transform for inshore ship detection, but its recall dropped by over 40% when ships were partially occluded. Li-Bing Jiang et al.’s [15] AIAC-based method suffered a 28% false detection rate in variable illumination conditions, while Hu et al. [16] and Shi et al. [17] reported significant performance degradation in open-sea or haze environments. These limitations made traditional methods unsuitable for real-world USV deployment.

With the breakthrough of CNN-based ImageNet classification [18], deep learning became the mainstream for sea surface detection, divided into two-stage (precision-focused) and single-stage (speed-focused) paradigms. Two-stage methods like RCNN [19] and Faster RCNN [20] achieved high precision but sacrificed speed—Faster RCNN reduced inference time to 0.5 s per image but still limited FPS to <50, incompatible with USV real-time requirements [21]. Extensions like Rotated R-CNN [22] improved orientation adaptation for slender ships but retained serial processing inefficiencies.

Single-stage methods were more suitable for edge deployment, with SSD and the YOLO series being representative. SSD [23] used multi-scale feature maps, but its fixed anchors failed to fit ships with extreme aspect ratios, leading to 15% missed detection [24]. The YOLO series [25] became dominant for its accuracy–speed balance: Li et al. [26] optimized it for thermal infrared images, Hu et al. [27] designed a lightweight variant for small ships, and Chen et al. [28] integrated attention for distant targets. Related works validated attention mechanisms (e.g., coupled attention architectures, which have shown strong fine-grained feature perception capability in target-related tasks [29,30]) and multi-task adaptability [31] of YOLO, while anchor-free detectors like CPS-Det [32] avoided anchor mismatch but increased computational cost. Yao et al. [33] laid the foundation by applying deep CNNs to ship detection, outperforming traditional methods by 12% mAP.

YOLOv8, with its optimized C2f Backbone and PAN-FPN Neck, is now the sea surface detection benchmark, but existing improvements focus on single dimensions. Zhao et al. [34] enhanced feature extraction with MobileViT and GSConv; Gong et al. [35] added cross-scale attention for long-distance targets, and continuous attention designs [36] have also demonstrated effectiveness in capturing persistent feature dependencies for target classification; Huang et al. [37] modified the loss function for SAR noise; and Feng et al. [38] used data augmentation for attitude variation. Even the latest YOLO11 struggles in complex scenarios, with mAP dropping by 6.3% in open-ocean SAR environments [39].

Despite the remarkable progress of YOLO-based methods in object detection, they still exhibit certain limitations when applied to complex marine scenarios. Firstly, in terms of multi-scale and asymmetric target adaptation, the 3 × 3 convolutional layers in YOLOv8, with their fixed receptive fields, tend to face challenges in capturing the contextual dependencies of small targets and accurately modeling the geometric characteristics of slender maritime targets. Secondly, regarding feature fusion, the static upsampling mechanism commonly used in existing YOLO architectures may lead to the loss of fine-grained edge information for small ships, which stands in contrast to the improved performance brought by dynamic feature fusion techniques [40]. Thirdly, the issue of regression stability for small targets remains to be addressed: the traditional CIoU loss function may show instability in bounding box regression for small maritime targets, and although related solutions have been explored in underwater target detection [41], this problem has not yet been effectively resolved for sea surface scenarios. Notably, few existing studies have systematically addressed these three interrelated challenges simultaneously.

To address these challenges, this paper proposes $M^{2}S\text{-}\mathrm{YOLOv}8$ based on YOLOv8, with three systematic enhancements:

- Asymmetric Multi-Scale Feature Extraction: The MSA-PPA block integrates parallel branches for local–global feature capture and asymmetric direction awareness, improving detection of small/occluded targets and slender vessels.
- Adaptive Feature Upsampling and Fusion: DCNUpsample replaces fixed interpolation, learning dynamic offsets to adapt to nonuniform sea surface features and enhance multi-scale fusion flexibility.
- Weighted Stable Localization Loss: WA-CIoU loss combines Wasserstein distance with CIoU, mitigating gradient imbalance to improve bounding box regression precision for small targets.

Designed for USV edge deployment, $M^{2}S\text{-}\mathrm{YOLOv}8$ provides a lightweight, real-time solution for environmental perception. Extensive experiments on marine datasets demonstrate its superior mAP@0.5 and FPS balance. Ablation studies confirm the MSA-PPA block’s contribution to asymmetric target perception and WA-CIoU’s role in stable small-target localization, validating the proposed design.

## 2. Methodology

### 2.1. $M^{\text{2}}S\text{-}\mathrm{YOLOv}\text{8}$ Architecture

**Figure 1:**
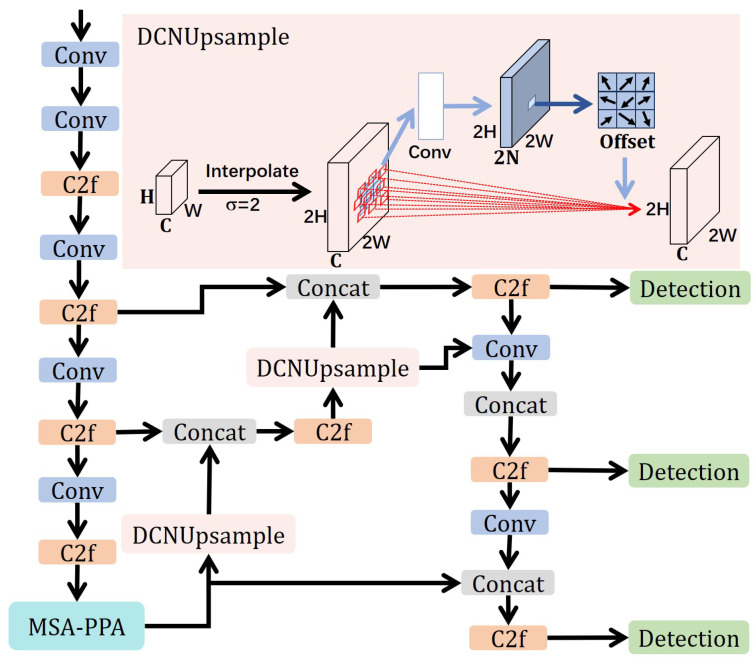
Overview architecture of $M^{2}S\text{-}\mathrm{YOLOv}8$, which adopts the MSA-PPA module and DCNUpsample.

As shown in Figure 1, the overall architecture of the sea surface target detection model proposed is based on the YOLOv8 model, primarily consisting of three main components: the Backbone feature extraction network, the Neck feature fusion network, and the Detection Head. Additionally, a multi-scale attention mechanism, deformable convolution upsampling operators, and an improved loss function are incorporated to enhance detection performance in complex marine environments. The overall process is as follows:

First, during the Backbone stage, lightweight convolutional neural networks are employed as the main architecture to extract features at different resolutions layer by layer, forming multi-scale feature representations. In this process, a Multi-Scale Asymmetric-aware Parallelized Patch-Aware Attention (MSA-PPA) block is embedded. This block employs parallel branch structures to model both local details and global context features. One branch utilizes local convolutions to capture fine-grained texture information, while the other branch acquires long-range dependencies by expanding the receptive field. Subsequently, the outputs of these branches are fused using channel-wise and spatial attention mechanisms, thereby integrating both local and global information in the feature representation. Additionally, the MSA-PPA block introduces an asymmetric direction perception mechanism in the convolution kernel design, enhancing the network’s sensitivity to target geometric shapes and orientations through asymmetric convolutions in horizontal and vertical directions. This adaptation allows the network to better accommodate the complex changes in scale, morphology, and orientation of sea surface targets.

During the Neck stage, the model employs an enhanced feature pyramid structure to fuse multi-scale features from the Backbone layer by layer, facilitating cross-level information interaction. Unlike traditional methods, this study introduces the Deformable Convolution Upsample Operator (DCNUpsample) during the upsampling process, replacing fixed bilinear or nearest neighbor interpolation. This operator achieves adaptive spatial sampling through learned offsets. DCNUpsample dynamically adjusts sampling positions based on feature distributions, thereby preserving the geometric structure and edge information of targets during feature fusion. This enhancement significantly improves the flexibility and robustness of feature representation. This improvement is particularly crucial for marine target detection, as marine targets often exhibit irregular scales, blurred edges, and varied shapes.

In the Head stage, the model performs regression predictions on features at different scales to cover objects of various sizes, including large, medium, and small. To further improve the localization accuracy of small objects, we propose a Weighted Normalized CIoU Loss Function based on Wasserstein Distance (WA-CIoU). This loss function introduces Wasserstein distance to measure the distributional differences between predicted and ground truth bounding boxes, based on the geometric constraints of traditional CIoU. Additionally, it employs a weighted normalization strategy to alleviate the issue of small gradients during the regression process of small objects. Through this design, the model achieves a more balanced gradient distribution in small object detection, thereby enhancing overall localization accuracy and stability.

In summary, the Backbone stage enhances the multi-scale and directional perception capabilities of feature extraction through the MSA-PPA block; the Neck stage improves the adaptability and expressiveness of feature fusion through DCNUpsample; and the Head stage optimizes the localization accuracy of small targets through the WA-CIoU loss function. This overall design significantly enhances the detection capability for multi-scale, asymmetric, and small targets in complex marine environments while ensuring model lightweighting and real-time performance, providing an efficient and reliable solution for surface target detection tasks.

### 2.2. MSA-PPA

The marine environment exhibits significant complexity. Under conditions of dense ship traffic, nighttime, overcast days, or adverse sea states, visible light images often feature cluttered backgrounds and low contrast between targets and the background. Additionally, marine targets, particularly distant ships, frequently appear as small-scale objects with substantial variations in size. More complexly, ships generally exhibit asymmetric geometric characteristics: cargo ships and oil tankers typically have a slender structure, whereas tugboats and patrol boats may appear nearly rectangular or short and wide. This asymmetry results in significant morphological differences in ship appearances under different viewing angles, orientations, and imaging distances. For instance, the long axis of the ship may be parallel or perpendicular to the sensor field of view, leading to pronounced changes in appearance. These factors render traditional target detection methods, which rely on fixed scales or symmetric assumptions, less effective. This underscores the necessity for enhanced multi-scale feature representation and direction perception capabilities in detection models. These phenomena pose significant challenges to the accuracy and robustness of marine target detection.

Despite the outstanding performance of YOLOv8 in general object detection tasks, its application in specific marine scenarios is still significantly limited. Despite the notable computational efficiency exhibited by YOLOv8, its core convolutional operations are inherently constrained to local receptive fields. This limitation impedes the effective modeling of long-range spatial dependencies between distant small targets and complex sea surface backgrounds. More critically, the design inherently relies on assumptions of target symmetry and fixed-scale priors, assuming that targets exhibit relatively uniform scale distributions and geometric symmetries in the spatial domain. This assumption fundamentally conflicts with the prevalent long-to-wide asymmetrical geometric characteristics of ship targets in marine environments.

The above mechanistic deficiencies result in YOLOv8’s inadequate capture of global contextual information and direction-sensitive features for small-scale and asymmetric geometric targets on the sea surface from long distances: On one hand, the model struggles to effectively perceive long-range dependencies between targets and backgrounds, leading to limitations in feature extraction and localization accuracy for small targets in complex marine environments with low contrast and significant scale variations, which can easily result in missed detections or false positives. On the other hand, its preconceived symmetry in target morphology makes it unable to adapt to the visual saliency differences in width-to-length directions caused by the nonsymmetric aspect ratios of ships, thereby failing to effectively distinguish key discriminative features under different orientations, further increasing the detection difficulty. This limitation reflects the inherent inadequacies of traditional convolutional neural networks in handling marine target detection tasks with long-range dependencies and direction sensitivity, and there is an urgent need to improve through the introduction of more effective multi-scale feature extraction strategies and direction-adaptive attention mechanisms.

Traditional global attention mechanisms, such as SE (Squeeze-and-Excitation) and CBAM (Convolutional Block Attention Module), although capable of enhancing the model’s focus on critical regions, suffer from high computational costs in global correlation calculations, particularly when processing high-resolution sea surface images. This makes it difficult to meet the real-time requirements of ocean monitoring applications. To reduce computational burdens, this study draws inspiration from the PPA (Parallelized Patch-Aware Attention) module in HCF-Net, which involves spatially partitioning patches and parallelly extracting local information to achieve significant improvements in computational efficiency. However, the original PPA module still falls short in capturing hierarchical dependencies across multiple scales, modeling asymmetric directional sensitivities, and semantic fusion capabilities, thereby limiting its target representation ability in complex ocean scenarios.

**Figure 2:**
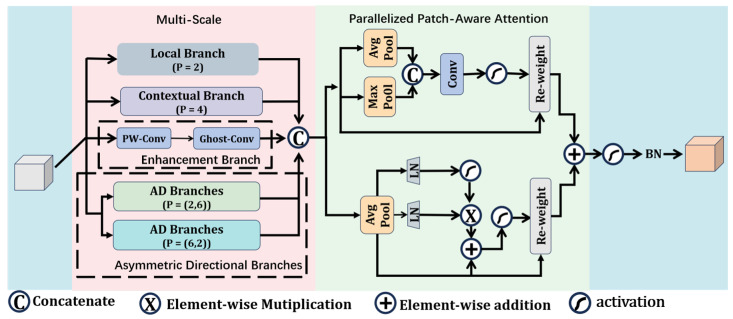
Structure of Multi-Scale Asymmetric-aware Parallelized Patch-Aware Attention (MSA-PPA), which employs parallel branch structures to model both local details and global context features.

To address the aforementioned issues, this paper designs the MSA-PPA block and integrates it into the SPPF layer of the YOLOv8 backbone network. As shown in Figure 2, MSA-PPA employs an efficient multi-branch structure to not only achieve comprehensive representation of multi-scale features but also enhance the model’s discriminative ability for asymmetric geometric features, thereby improving the model’s perception capability and detection accuracy for small targets on the sea surface. This module provides strong support for ship detection in complex marine scenarios. As shown in the figure, the MSA-PPA module adopts a parallel multi-branch structure, mainly consisting of a local branch, a context branch, an enhancement branch, and asymmetric directional branches.

Local Branch: This branch employs a small patch division strategy with a size of p = 2, focusing on extracting fine local features of the target. This enhances the model’s ability to depict details such as edges, contours, and textures of small targets. In scenarios with densely packed ships or cluttered backgrounds, this branch can effectively distinguish targets from nearby distractions, improving the feature recognition of small targets and providing crucial local discriminative evidence for subsequent detection.

Contextual Branch: This branch employs patches with a larger receptive field (p = 4) to model long-range spatial dependencies between targets and their surrounding environment. By covering a larger receptive field, the module can perceive the overall semantic associations between targets and backgrounds (e.g., the relative positions of ships and waves, or clouds), which aids the model in understanding the overall semantic structure of targets in relation to their environment. This facilitates accurate localization of targets in complex backgrounds.

Enhancement Branch: By combining pointwise convolution (PW-Conv) and Ghost-Conv in series, further supplementary cross-channel semantic information is provided. In traditional convolution modules, although PW-Conv can achieve linear interactions between channels with extremely low computational cost, its capability in expressing nonlinear features is limited. Ghost-Conv, on the other hand, employs a strategy of base feature generation plus inexpensive linear transformation to generate diverse ghost features while reducing computational load, thereby compensating for the information loss in the original features.

Asymmetric Directional Branches (p = (2,6) and p = (6,2)): To adapt to the typical visual characteristics of ship targets in marine scenarios and the background interference characteristics, this paper innovatively introduces two asymmetric patch division branches—p = (2,6) branch and p = (6,2) branch. The design principle is based on the following interpretability analysis:

p = (2,6) branch: This branch employs a fine-grained patch division strategy in the horizontal direction (p = 2) and a coarse-grained strategy in the vertical direction (p = 6). Far-distance ships typically exhibit horizontal extension features, while background interference often has dynamic changes in the vertical direction. This branch achieves precise capture of the horizontal edges and structural details of the ship through fine-grained perception in the horizontal direction. Meanwhile, coarse-grained processing in the vertical direction effectively suppresses irrelevant vertical texture noise, enhancing attention to the main structure of the ship.

p = (6,2) branch: This branch complements the p = (2,6) branch by using a fine-grained patch division strategy in the vertical direction (p = 2) and a coarse-grained strategy in the horizontal direction (p = 6). This design focuses on extracting vertical structural features of the ship, and through coarse-grained modeling in the horizontal direction, it constructs a broader range of scene context associations.

The two branches work collaboratively to achieve complementary perception of structural information from multiple directions, enhancing the model’s adaptability to ships in different orientations (e.g., tilting, turning).

Each parallel branch outputs local detail features Xlocal, global context features Xcontext, cross-layer enhanced features Xenhance, horizontal asymmetric features X(2,6), and vertical asymmetric features X(6,2). Subsequently, these features are concatenated (Concat) along the channel dimension to generate a fused feature representation Xfused, thereby providing more comprehensive and diverse feature inputs for subsequent specialized fusion.

(1) $$X_{fused}=Concat\left(X_{local},X_{context},X_{enhance},X\left(2,6\right),X\left(6,2\right)\right)$$

To extract key information from the fused feature $X_{fused}$, MSA-PPA introduces a parallel dual-path structure comprising a channel attention branch and a spatial attention branch. These branches dynamically weight features from the perspectives of channel importance and spatial location importance, respectively, and ultimately achieve multi-dimensional attention fusion through feature summation. The output is the enhanced feature $X_{out}\in R^{C\times H\times W}$. This design explicitly applies dual-dimensional attention weighting to suppress background noise and enhance discriminative target features, thereby providing more distinguishable inputs to subsequent detection heads.

The channel attention branch aims to enhance the channel representations that are discriminative for target detection, particularly for small-scale vessels, while suppressing irrelevant channel interference introduced by complex sea surface backgrounds. Specifically, the spatial dimensions (H × W) of $X_{fused}$ are first globally averaged pooled (AvgPool) to compress each channel’s two-dimensional features into scalars, resulting in a channel-level global description $X_{avg}\in R^{C\times 1\times 1}$. This operation effectively reduces the interference of local variations on the assessment of channel importance by aggregating global spatial information, enabling the model to focus on the overall response strength of channels.

During the channel attention reconstruction phase, this study employs the Gated Linear Unit (GLU) to enhance nonlinear modeling capabilities and achieve more flexible information selection. Specifically, $X_{avg}$ is first mapped through two independent linear transformations to obtain baseline feature vectors and gate feature vectors. Subsequently, the gate feature vectors are normalized to the [0, 1] interval using the GELU activation function σ(·), resulting in channel attention weights $W_{c}\in R^{C\times 1\times 1}$, where the magnitude of each value reflects the importance of the corresponding channel for target detection. Finally, $W_{c}$ is element-wise multiplied with the original feature $X_{fused}$ to generate channel attention enhanced features.

(2) $$\begin{array}{cc} X_{avg} & =\mathrm{AvgPool}(X_{fused}) \end{array}$$

(3) $$\begin{array}{cc} X_{ch} & =W_{3}(\left(W_{1}\cdot X_{avg}\right)⊙\sigma \left({\mathrm{DWConv}}_{3\times 3}\left(W_{2}\cdot X_{avg}\right)\right)+X_{avg} \end{array}$$

This process dynamically weights to enhance the responses of discriminative channels for ship edges and profiles, while effectively suppressing background interference channels such as waves and clouds, thereby improving the detectability of small-scale targets in complex marine environments.

The spatial attention branch is designed to guide the model to focus on significant areas in the spatial dimension that are relevant to the ship target (such as the hull contour and mast structure), while suppressing interference from complex oceanic backgrounds (such as waves, clouds, and reflection spots). To achieve this, the branch integrates multi-scale spatial statistical features with lightweight convolutional mappings to generate a spatial weight matrix $W_{s}$. For the fused feature map $X_{fused}$, global maximum pooling (MaxPool) and global average pooling (AvgPool) are applied separately to each spatial location, obtaining the maximum and average response values, respectively. The former highlights the saliency responses of the target area, while the latter provides a global description of the background distribution. These two values are then concatenated along the channel dimension to form a composite spatial description:

(4) $$X_{spa}\;=\;\left[\mathrm{MaxPool}\left(X_{fused}\right);\mathrm{AvgPool}\left(X_{fused}\right)\right]\in R^{2\times H\times W}$$

To achieve the integration and compression of spatial information, a 1×1 convolution is applied to $X_{spa}$, and the output is normalized to the range [0, 1] using the Sigmoid activation function σ(·), thereby obtaining the spatial attention weight matrix.

(5) $$W_{s}\;=\;\sigma \left({\mathrm{Conv}}_{1\times 1}\left(X_{spa}\right)\right)\in R^{1\times H\times W}$$

Here, the weight values reflect the importance of the corresponding spatial positions for target detection; the higher the value, the more likely the position corresponds to a significant area of the ship.

Finally, the weight matrix $W_{s}$ is element-wise multiplied with the original fused features $X_{fused}$ along the spatial dimension, resulting in the spatially enhanced attention features.

(6) $$X_{s}\;=\;W_{s}⊗X_{fused}\in R^{C\times H\times W}$$

Through this mechanism, the model can adaptively highlight the spatial responses of the main body of ships in complex marine scenarios while effectively suppressing interference in the background areas, thereby enhancing the sensitivity and robustness of detection for small-scale ships. To integrate attention information from both the channel and spatial dimensions, the module adds, element-wise, the channel attention feature $X_{c}$ and the spatial attention feature $X_{s}$, fusing multi-dimensional attention gains. Subsequently, a batch normalization (BN) layer stabilizes the feature distribution, and a rectified linear unit (ReLU) activation function introduces nonlinearity. Finally, the output feature of the MSA-PPA module, $X_{out}\in R^{C\times H\times W}$, is generated.

(7) $$X_{out}=\mathrm{BN}\left(\mathrm{ReLU}\left(X_{c}+X_{s}\right)\right)$$

### 2.3. DCNUpsample

In ship detection tasks on the sea surface, the inherent significant scale differences of targets and complex background interference pose severe challenges to feature representation. The original Neck of YOLOv8 employs a feature pyramid structure to fuse shallow high-resolution features with deep semantic features, thereby enhancing multi-scale object detection performance. It uses traditional bilinear interpolation for upsampling, which, although computationally efficient, employs a fixed, content-agnostic sampling strategy that struggles to effectively preserve critical edge and texture information when handling distant small-scale ships. This often results in feature blurring and loss of spatial details, thereby limiting the model’s detection accuracy.

(8) $$X_{up}\;=\;\mathrm{Interp}\left(X,s\right)$$

where Interp(·) denotes the static interpolation function, and *s* is the scale factor. This method relies solely on rule-based sampling and has difficulty preserving the geometric details of complex targets.

To address this limitation, as shown in Figure 2, we introduce the Deformable Convolution Upsampling Operator (DCNUpsample) into the Neck of YOLOv8, replacing static interpolation methods to enhance the adaptability and expressiveness of feature fusion. The core idea of DCNUpsample is to introduce learnable sampling offsets, allowing the convolution kernel to dynamically adjust the sampling positions on the feature map, thus achieving more flexible feature reconstruction. Specifically, for an output position $p_{0}$, its feature value is given by the following equation:

(9) $$\Delta P=f_{\theta }\left(X_{up}\right),\Delta P\in R^{2K\times H^{\prime }\times W^{\prime }}$$

where $f_{\theta }\left(\cdot \right)$ denotes the offset prediction convolution, *K* is the number of sampling points in the convolution kernel (e.g., K=9), and $\left(H^{\prime },W^{\prime }\right)$ is the resolution after upsampling.

Next, adaptive sampling of features is performed through deformable convolutions:

(10) $$X^{\prime }\left(p_{0}\right)=\sum_{k=1}^{K}w_{k}\cdot X\left(p_{0}+p_{k}+\Delta p_{k}\right)$$

where $p_{k}$ represents the regular sampling points of the convolution kernel, $\Delta p_{k}$ is the offset predicted by the input feature, and $w_{k}$ is the weight of the convolution kernel. Compared to traditional convolution, the offset $\Delta p_{k}$ allows the convolution kernel to flexibly select optimal sampling positions in space, thereby better characterizing the edges and detailed features of ship targets in complex backgrounds.

### 2.4. WA-CIoU: Loss Function Optimization

In marine surface datasets, distant ships, buoys, and other small targets are commonly present. These targets typically provide minimal visual information, significantly increasing the difficulty of accurately identifying and classifying them. The original boundary box loss function in YOLOv8 consists of two parts: classification loss and regression loss. The classification loss is computed using binary cross-entropy (BCE), while the regression loss is computed using CIoU + DFL. Although traditional CIoU loss improves the IoU metric by incorporating constraints on the center point distance and aspect ratio, it still has inherent limitations when dealing with small targets. First, when the target size is smaller than 32×32 pixels, even minor shifts in the boundary box can cause the IoU value to fluctuate dramatically, leading to unstable gradient updates during backpropagation. Second, existing methods rely solely on geometric features for bounding box regression, neglecting the spatial characteristics of the target’s internal pixel distribution.

To address these issues, this study integrates the Wasserstein distance from optimal transport theory to propose a novel loss function, WA-CIoU, specifically optimized for small target detection. WA-CIoU aims to balance the detection performance of both small and large targets, ensuring that the model can stably recognize objects of various sizes on the marine surface. The WA-CIoU loss function is defined as follows:

First, the boundary boxes of small targets usually contain not only foreground pixels but also a significant number of background pixels. Foreground pixels are primarily concentrated in the central region, while background pixels are more prevalent at the edges. To effectively represent the relative importance of each pixel within the boundary box, this study models the boundary box using a two-dimensional Gaussian distribution. Specifically, given a boundary box $R=(c_{x},c_{y},w,h)$, where $\left(c_{x},c_{y}\right)$, *w* and *h* represent the center coordinates, width, and height, respectively, it is mapped to a two-dimensional Gaussian distribution N(μ,Σ). Here, the mean vector $\mu =(c_{x},c_{y})$ and the covariance matrix Σ are determined by the dimensions of the boundary box, where the weights decay gradually from the center to the edges, thereby quantifying the degree of match between the detection box and the ground truth box through the differences in statistical properties of the distributions. For a predicted box $P∼N(\mu_{p},\Sigma_{p})$ and a ground truth box $G∼N(\mu_{g},\Sigma_{g})$, the Wasserstein distance from optimal transport theory is used to compute the distribution distance, with the second-order Wasserstein distance given as follows:

(11) $$W_{2}^{2}\left(N_{p},N_{g}\right)\;=\;{∥\begin{array}{c} \left(\begin{array}{c} {\left[cx_{p},cy_{p},\frac{w_{p}}{2},\frac{h_{p}}{2}\right]}^{T},{\left[\begin{array}{c} cx_{g},cy_{g},\frac{w_{g}}{2},\frac{h_{g}}{2} \end{array}\right]}^{T} \end{array}\right) \end{array}∥}_{2}^{2}$$

where $∥\cdot ∥$ represents the Frobenius norm.

Furthermore, as the aforementioned equation belongs to a distance metric, it cannot be used directly as a measure of similarity. Therefore, it requires an exponential transformation for normalization processing, thereby obtaining a new metric, namely, the normalized Wasserstein distance (NWD), as follows:

(12) $$NWD\left(N_{a},N_{b}\right)\;=\;exp\left(\begin{array}{c} -\frac{\sqrt{W_{2}^{2}\left(N_{a},N_{b}\right)}}{C} \end{array}\right)$$

where the normalization constant *C* is dataset-specific and determined by the statistical average size of all ground truth boxes. If only the NWD metric is used as the localization loss function for the model, it may not achieve the best detection performance for multi-scale datasets and can also lead to a slower convergence of model training. Therefore, to balance the contribution of the NWD loss function and its convergence issues, this paper introduces a scaling factor μ to dynamically adjust the weight of the NWD loss function, thereby optimizing detection performance. The new loss function is defined as WA-CIoU:

(13) $$L_{WA-CIoU}\;=\;\mu L_{CIoU}+\left(1-\mu \right)L_{\mathrm{NWD}}$$

Overall, WA-CIOU alleviates the inherent gradient instability issues encountered by IoU-based metric methods when dealing with small-scale targets by reconfiguring the regression problem from a traditional geometric overlap perspective into a probabilistic distribution matching framework. Gaussian modeling, as an effective spatial prior, guides the model to align with the salient core regions of the target rather than its complete bounding box, thereby enhancing localization accuracy by reducing sensitivity to background noise. Additionally, its hybrid integration strategy with traditional CIoU loss ensures a balanced and comprehensive optimization across a wide range of target scales, thereby improving the performance of small targets without compromising the detection accuracy of larger targets. Consequently, WA-CIoU provides a more robust, stable, and spatially aware optimization objective that can effectively enhance recall and precision in complex maritime surveillance scenarios, particularly for distant vessels and buoys that are small but critical.

## 3. Model Comparison

### 3.1. Datasets

To comprehensively evaluate the adaptability and robustness of the proposed method across diverse marine scenarios, multiple benchmark datasets were employed to conduct ship target detection and tracking experiments, including the Unmanned Vessel Zhoushan Perception Dataset (UZPD) and the open-source Singapore Maritime Dataset (SMD) [42].

**Figure 3:**
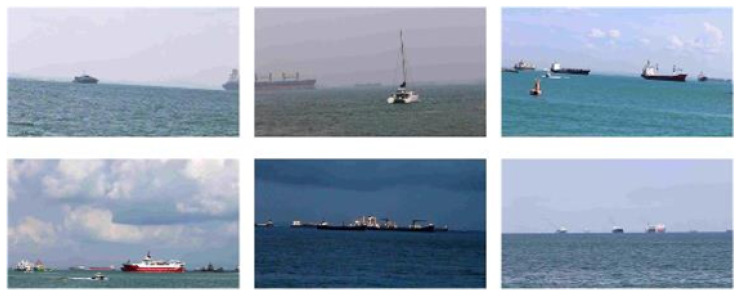
Singapore Maritime Dataset. It covers both shore-based and ship-borne observation scenarios.

As shown in Figure 3, the SMD was developed by the Rolls-Royce Laboratory at Nanyang Technological University, encompassing core tasks such as ship segmentation, inspection, and tracking. The image data were captured offshore using a Canon 70D camera with a resolution of 1080 × 1920 pixels, covering both shore-based and ship-borne observation scenarios. The dataset consists of 81 video sequences recorded over a time span from July 2015 to May 2016, which systematically includes various challenging environmental conditions: pre-sunrise, noon, afternoon, evening, post-sunset, haze, and rainfall. For the purpose of this study, the video sequences were sampled and preprocessed, ultimately yielding 8457 static images for model training and evaluation.

**Figure 4:**
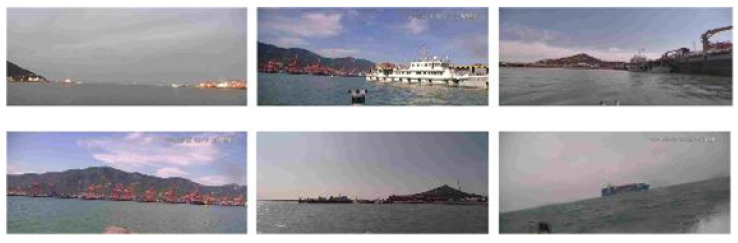
Unmanned Vessel Zhoushan Perception Dataset provided by CETC MaritimeElectronics Ltd.

The UZPD is a large-scale dedicated dataset tailored for maritime perception and target detection research. It is provided by CETC Maritime Electronics Ltd. and the data was collected on board unmanned surface vessels. It focuses on five typical categories of maritime targets: cargo ships, fishing boats, speedboats, buoys, and coast guard vessels displayed in Figure 4. The dataset covers a wide range of realistic maritime scenarios, including port areas, outbound shipping lanes, inbound shipping lanes, and open offshore regions, thus providing sufficient scene diversity for performance validation.

For a consistent experimental setup, both the SMD and UZPD were randomly partitioned into training, validation, and test sets following a unified split ratio of 7:2:1. This random division strategy ensures sufficient training data to facilitate model convergence, while reserving representative and unbiased samples for objective performance evaluation.

### 3.2. Experimental Setup

All experiments were conducted on a workstation equipped with an AMD EPYC 9754 CPU (Advanced Micro Devices, Inc., Santa Clara, CA, USA) and an NVIDIA GeForce RTX 3090 GPU (NVIDIA Corporation, Santa Clara, CA, USA); and the software environment included Python 3.8.10, PyTorch 2.0.0, and CUDA 11.8; the key training hyperparameters were configured as follows: momentum of 0.937, initial learning rate of 0.01, batch size of 16, SGD optimizer, total training epochs of 200, and weight decay of 0.005. Additionally, the data augmentation strategies inherent to YOLOv8 were adopted, including random scaling, random cropping, Mosaic augmentation, HSV color space adjustment, and Gaussian blur.

### 3.3. Evaluation Metrics

To comprehensively evaluate the overall performance of the $M^{2}S\text{-}\mathrm{YOLOv}8$ model in target detection tasks, this paper adopted multiple mainstream evaluation metrics, including mean average precision (mAP@50), number of parameters (Params), frames per second (FPS), and floating point operations per second (FLOPs). The calculations for precision, recall, and mean average precision are all based on the results from the confusion matrix, as shown in Table 1.

TP (True Positives) denotes the number of positive samples correctly classified as positive, FN (False Negatives) denotes the number of positive instances incorrectly classified as negative, FP (False Positives) denotes the instances where negative samples are incorrectly classified as positive, and TN (True Negatives) denotes the number of negative instances correctly classified by the classifier.

#### 3.3.1. Precision

Precision is the proportion of true positive cases among the samples predicted as positive:

(14) $$\mathrm{Precision}\;=\;\frac{\mathrm{TP}}{\mathrm{TP}+\mathrm{FP}}$$

#### 3.3.2. Recall

Recall reflects the model’s ability to identify true positive samples, specifically the proportion of correctly detected positive samples among all true positive samples:

(15) $$\mathrm{Recall}\;=\;\frac{\mathrm{TP}}{\mathrm{TP}+\mathrm{FN}}$$

#### 3.3.3. Mean Average Precision

Mean average precision, mAP for short, is a widely used comprehensive evaluation metric in the field of object detection. It reflects the average performance of the model at different recall levels by calculating the area under the precision–recall curve. First, the average precision (AP) for each category is computed, and then the average of these AP values across all categories is taken to obtain the overall mAP metric. This metric can be used not only for single-class performance analysis but also for horizontal comparisons between models.

(16) $$\begin{array}{cc} \mathrm{AP} & =\sum_{n=1}^{N}\left(R_{n}-R_{n-1}\right)P_{n} \end{array}$$

(17) $$\begin{array}{cc} \mathrm{mAP} & =\frac{1}{C}\sum_{c=1}^{C}{\mathrm{AP}}_{c} \end{array}$$

where $P_{n}$ and $R_{n}$ are the precision and recall corresponding to the nth threshold, respectively; *N* is the number of thresholds; *C* is the number of categories; and ${\mathrm{AP}}_{c}$ is the AP value for the cth category.

#### 3.3.4. FLOPs

FLOPs serve as an important reference metric for assessing the computational overhead of a model, representing the total number of floating point operations required to complete a forward inference. Lower FLOPs indicate higher computational efficiency, making the model particularly suitable for deployment on devices with limited computational power. Reducing FLOPs also aids in decreasing energy consumption and enhancing inference speed.

#### 3.3.5. Params

Params refers to the total number of parameters that need to be learned during training, serving as an important metric for assessing model complexity and storage overhead. A smaller parameter count helps in reducing the model size and lowering memory consumption, and it also enhances inference speed and operational efficiency during deployment.

#### 3.3.6. FPS

FPS measures the number of image frames a model can process within a unit of time. For tasks with high real-time requirements (such as video stream detection and intelligent surveillance), a higher FPS indicates that the model possesses stronger online processing capabilities.

### 3.4. Experimental Results and Analysis

To comprehensively evaluate the overall performance of the proposed $M^{2}S\text{-}\mathrm{YOLOv}8$ model in marine surface object detection tasks, this section selects various representative object detection algorithms for comparison, including two-stage detection models (Faster R-CNN), single-stage detection models from the YOLO series (YOLOv5s, YOLOv9s, YOLOv10s, YOLOv11s, YOLOv12s), and transformer-based detectors (RT-DETR-L).

As shown in Table 2, general-purpose object detection algorithms (e.g., Faster R-CNN and RT-DETR-L) exhibit substantially higher parameter counts and computational complexity compared to lightweight models, translating to slower detection speeds (46.2 and 35.7 FPS, respectively). This renders them unable to meet the real-time requirements for target detection tasks on unmanned surface vehicles (USVs). In contrast, lightweight YOLO-series algorithms possess distinct advantages in model compression and detection efficiency. Our $M^{2}S\text{-}\mathrm{YOLOv}8$ achieves a balanced performance between lightweight design and detection accuracy through network structure optimization: while it is comparable to other YOLO variants in terms of computational complexity and detection speed, it offers a significant improvement in detection accuracy.

**Figure 5:**
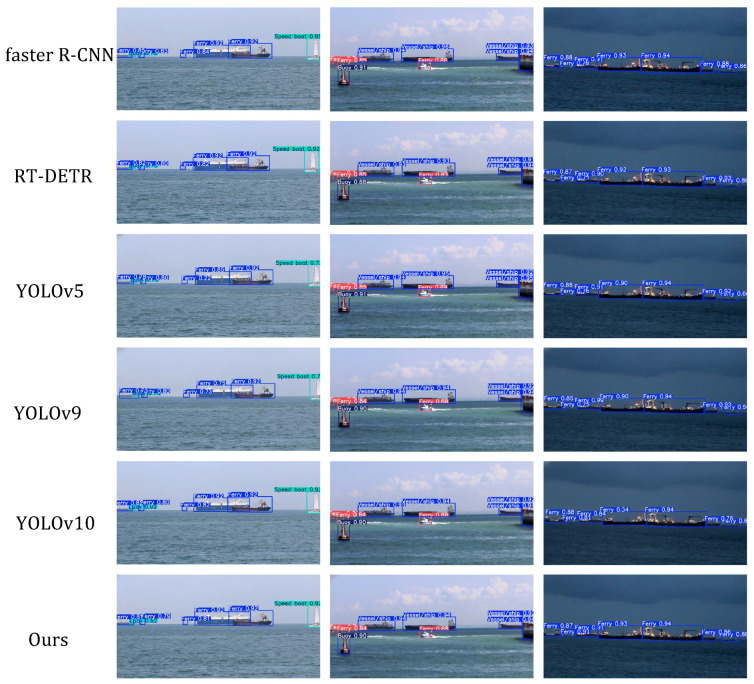
Comparative visualization of detection results across different models. From top to bottom, the results correspond to Faster R-CNN, RT-DETR, YOLOv5, YOLOv9, YOLOv10, and our $M^{2}S\text{-}\mathrm{YOLOv}8$.

Specifically, YOLOv9s features the lowest parameter count, while YOLOv5s delivers the optimal FLOPs and FPS across all compared algorithms; our $M^{2}S\text{-}\mathrm{YOLOv}8$, however, attains the highest mAP, realizing an optimized trade-off among lightweight properties, detection accuracy, and inference speed. Compared to baseline algorithms, $M^{2}S\text{-}\mathrm{YOLOv}8$ further enhances accuracy while guaranteeing real-time detection—making it particularly suitable for marine target detection scenarios where real-time performance and resource efficiency are critical. Visual comparison results of the different algorithms are presented in Figure 5.

Figure 5 presents the results of contrast experiments involving different algorithms, from top to bottom, corresponding to the visualization detection results of Faster R-CNN, RT-DETR, YOLOv5, YOLOv9, YOLOv10, and our $M^{2}S\text{-}\mathrm{YOLOv}8$. It can be observed that our $M^{2}S\text{-}\mathrm{YOLOv}8$ performs more excellently in marine target detection tasks, particularly in handling occlusions, small targets, and distant targets, where the detection boxes are more precise. This fully demonstrates the advantages and effectiveness of $M^{2}S\text{-}\mathrm{YOLOv}8$ in complex marine scenarios.

### 3.5. Ablation Experiments

To quantify the contribution of each improved module to $M^{2}S\text{-}\mathrm{YOLOv}8$, we designed four groups of ablation experiments, with results summarized in Table 3. As shown, all proposed blocks enhance YOLOv8’s performance—particularly for small targets—on both maritime datasets, with distinct characteristics.

Specifically, the MSA-PPA integrates an asymmetric direction-aware mechanism in feature extraction, enhancing the network’s ability to capture multi-scale and oriented features in complex marine backgrounds. This boosts mAP by 2.2% (UZPD) and 2.3% (SMD), though the increased parameters/FLOPs slightly reduce FPS (139.1/137.4). The DCNUpsample replaces static interpolation in the Neck, dynamically adjusting sampling positions to reduce feature loss; it improves mAP by 1.3% (UZPD) and 1.2% (SMD) with minimal overhead, maintaining high real-time performance (141.4/140.2). The WA-CIoU loss alleviates traditional CIoU’s regression instability by introducing Wasserstein distance, mitigating IoU oscillations during gradient propagation. It increases mAP by 1.2% (UZPD) and 1.6% (SMD) without speed degradation. When integrated, $M^{2}S\text{-}\mathrm{YOLOv}8$ achieves optimal performance: 73.4% mAP (UZPD) and 71.9% mAP (SMD), a 3.4 percentage point improvement over the baseline. Despite the increased computational overhead, the model’s FPS (137.3/135.8) remains sufficient for real-time detection, which validates the synergistic effect of the proposed modules in balancing detection accuracy and real-time performance.

### 3.6. Impact of Factor μ in WA-CLoU

To explore the influence of the weight coefficient μ on the performance of the WA-CIoU loss function, four groups of controlled experiments were designed, where μ was configured as 0.2, 0.3, 0.4, and 0.5, respectively. By adjusting μ to these distinct values, we evaluated the model’s detection performance when incorporating the WA-CIoU loss function. Table 4 presents the model’s detection performance on the UZPD under these different parameter configurations.

As presented in Table 4, different values of μ exert a notable influence on the performance of $M^{2}S\text{-}\mathrm{YOLOv}8$ in sea surface object detection tasks. According to the experimental results, when μ is set to 0.3, the model attains the optimal performance in both mAP50 and mAP50-90 metrics, corresponding to a detection accuracy of 73.4%. Consequently, μ=0.3 is selected as the optimal configuration for subsequent experimental validation in this study. The WA-CIoU loss encodes target size information via the covariance matrix of a Gaussian distribution, which renders the similarity metric insensitive to absolute scale variations. This characteristic effectively mitigates the gradient imbalance issue encountered in small object detection. Compared with traditional IoU-based loss functions, WA-CIoU, thus, exhibits greater stability in detecting distant small objects on the sea surface.

### 3.7. Visualization

To improve the interpretability and visualization performance of the $M^{2}S\text{-}\mathrm{YOLOv}8$ object detection model, this study adopts the EigenCAM technique to visualize the internal feature activation of the model. Different from traditional gradient-based visualization methods, EigenCAM eliminates reliance on class-specific gradient information; instead, it performs eigenvalue decomposition on the feature maps output by convolutional layers to capture the dominant activation directions, thereby generating class-agnostic visualization heatmaps. Specifically, the last convolutional layer of the network was chosen as the key analysis object, and visualization experiments were conducted on the UZPD. The implementation process is as follows: first, the feature covariance matrix of the target layer’s feature maps was computed, followed by eigenvalue decomposition to extract eigenvectors corresponding to the main activation features; subsequently, activation maps were constructed based on the principal feature channels, which were then mapped back to the original input image spatial dimension to generate high-contrast heatmaps with intuitive interpretability.

**Figure 6:**
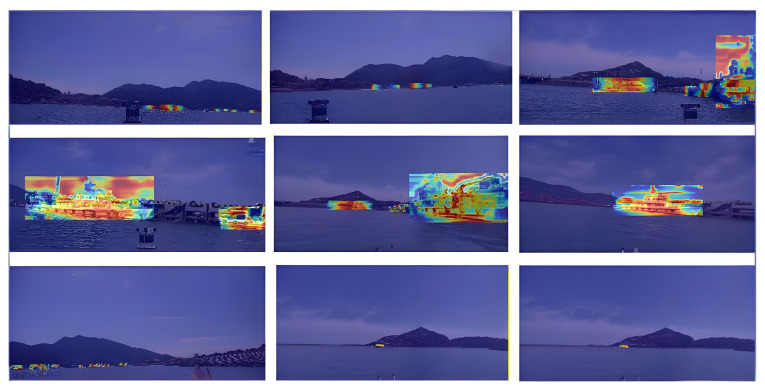
EigenCAM-based feature activation heatmaps of $M^{2}S\text{-}\mathrm{YOLOv}8$ in marine detection scenarios, where the colorful overlaid heatmaps reflect the regions focused on by the model during detection. These visualizations verify that $M^{2}S\text{-}\mathrm{YOLOv}8$ effectively concentrates attention on maritime vessel targets.

The visualization results obtained via EigenCAM are presented in Figure 6. As illustrated in the figure, the proposed $M^{2}S\text{-}\mathrm{YOLOv}8$ demonstrates a more concentrated focus on target-related regions during detection: the model’s attention is prominently aggregated at positions highly relevant to ship recognition, while background interference is effectively suppressed. This observation verifies that the improved algorithm possesses excellent feature discriminability and detection robustness.

### 3.8. Comparison with YOLOv8

**Figure 7:**
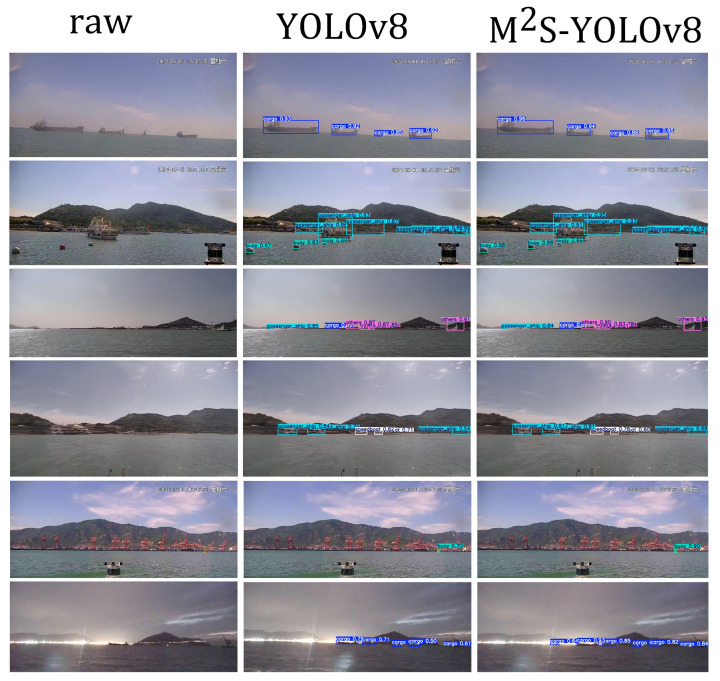
Comparison of detection performance improvements: The first column corresponds to the original images, the second column displays the detection results of YOLOv8, and the third column presents the detection results of $M^{2}S\text{-}\mathrm{YOLOv}8$. The Chinese meaning in the upper right corner of each picture is Saturday.

To verify the practical detection performance of the improved algorithm, this paper compares the baseline model with the enhanced $M^{2}S\text{-}\mathrm{YOLOv}8$ on different typical scenarios of the UZPD, as shown in Figure 7. In the first group of heavily foggy sea surface scenes, $M^{2}S\text{-}\mathrm{YOLOv}8$ significantly outperforms the baseline model in detection accuracy, enabling more stable identification of distant ship targets. In the second group of near-shore scenes with a sky background, due to less background interference, $M^{2}S\text{-}\mathrm{YOLOv}8$ can quickly and accurately locate maritime law enforcement ships and buoy targets with high confidence. Additionally, it demonstrates higher accuracy in small target annotation. In the third to fifth groups of scenes with overlapping multiple targets or complex backgrounds, despite the environmental complexity impacting detection accuracy, the performance of the improved algorithm remains significantly better than that of the baseline model: not only does it comprehensively detect ship targets and avoid missed detections, but it also shows a notable improvement in the identification accuracy of buoys and other small targets. In the fifth group of near-shore scenes with a cluttered background, buoys are still accurately detected with high confidence. In the sixth group under low-light nighttime conditions, the baseline model misses a cargo ship on the left side of the image, whereas $M^{2}S\text{-}\mathrm{YOLOv}8$ successfully detects this target based on its stronger feature representation capability, significantly enhancing nighttime detection stability. Overall, $M^{2}S\text{-}\mathrm{YOLOv}8$ exhibits excellent detection performance across various lighting conditions, complex backgrounds, and multi-scale target scenarios. It maintains the lightweight characteristic of the model while achieving high detection accuracy and stability. This indicates that this method has strong applicability and practical value in maritime ship target detection tasks.

## 4. Conclusions

To address the issues of sea surface vessel occlusion, small targets, and asymmetric structures, this study proposes $M^{2}S\text{-}\mathrm{YOLOv}8$ based on YOLOv8 for sea surface vessel detection. Specifically, by introducing the MSA-PPA module into the backbone, the acquisition of global and local features, as well as asymmetric perception, is realized, which enhances the detection performance for small, slender, and occluded vessels. By introducing DCNUpsample in the Neck to replace fixed interpolation, the model adapts to noncorresponding features and improves the flexibility of multi-scale fusion. By adopting the WA-CIoU loss to stabilize gradients, the gradient imbalance problem during training is alleviated. Through these optimization strategies, $M^{2}S\text{-}\mathrm{YOLOv}8$ delivers competitive performance on the SMD and UZPD. Although its FPS shows a slight decrease compared with the original YOLOv8, its mAP has been effectively improved.

However, $M^{2}S\text{-}\mathrm{YOLOv}8$ still has certain limitations. The detection stability of the model under extreme harsh weather conditions remains to be fully verified. Meanwhile, the current datasets may lack coverage of low-visibility scenarios, which may lead to performance limitations when the model relies solely on visible light images for detection. In the future, we plan to explore multi-modal data fusion technologies, such as fusing visible light, infrared, and synthetic aperture radar (SAR) data, to further mitigate the constraints brought by adverse environmental conditions.

## Figures and Tables

**Table 1:** Confusion matrix.

Reference	Prediction	
	Positive	Negative
Positive	TP	FN
Negative	FP	TN

**Table 2:** Comparison of different models.

Model	Params (M)	FLOPs (G)	UZPD		SMD	
			mAP (%)	FPS	mAP (%)	FPS
Faster-RCNN	31.2	126.4	61.7	46.2	60.3	45.8
YOLOv5s	7.2	16.4	64.8	165.4	63.9	162.7
YOLOv8s	11.2	28.6	70.0	144.1	68.5	142.7
YOLOv9s	7.1	26.7	68.2	146.4	67.4	145.0
YOLOv10s	7.2	21.6	70.1	148.5	69.2	147.1
YOLOv11s	9.4	21.5	70.3	148.8	69.5	147.5
YOLOv12s	9.3	21.4	70.6	150.2	69.8	148.7
RT-DETR-L	35.7	102.9	71.6	35.7	70.9	34.9
$M^{2}S\text{-}\mathrm{YOLOv}8$	13.9	36.3	73.4	137.3	71.9	135.8

**Table 3:** Ablation experiments on MSA-PPA, DCNUpsample, and WA-CIoU.

Model	Params (M)	FLOPs (G)	UZPD		SMD	
			mAP (%)	FPS	mAP (%)	FPS
YOLOv8 (baseline)	11.2	28.6	70.0	144.1	68.5	142.7
+MSA-PPA	13.8	35.7	72.2	139.1	70.8	137.4
+DCNUpsample	11.0	28.5	71.3	141.4	69.7	140.2
+WA-CIoU	11.2	28.7	71.2	144.2	70.1	143.3
$M^{2}S\text{-}\mathrm{YOLOv}8$ (All)	13.9	36.3	73.4	137.3	71.9	135.8

**Table 4:** Studies on different μ in WA-CLoU.

μ	UZPD		SMD	
	mAP50 (%)	mAP50-90 (%)	mAP50 (%)	mAP50-90 (%)
0.2	71.8	48.5	70.3	46.9
0.3	73.4	57.5	72.1	55.8
0.4	70.7	51.9	69.8	50.2
0.5	69.4	46.2	68.1	44.7

## Data Availability

The data that support the findings of this study are available from the corresponding author upon reasonable request.

## References

[1] Xiao G., Wang Y., Wu R., Li J., Cai Z. (2024). Sustainable maritime transport: A review of intelligent shipping technology and green port construction applications. J. Mar. Sci. Eng..

[2] Zou Y., Xiao G., Li Q., Biancardo S.A. (2025). Intelligent Maritime Shipping: A Bibliometric Analysis of Internet Technologies and Automated Port Infrastructure Applications. J. Mar. Sci. Eng..

[3] Kalinichenko Y., Rudenko S., Holovan A., Vasalatii N., Zaiets A., Koliesnik O., Santana L.O., Dolynska N. (2025). Smart Routing for Sustainable Shipping: A Review of Trajectory Optimization Approaches in Waterborne Transport. Sustainability.

[4] Ahammed S., Rana M.M., Uddin H., Majumder S.C., Shaha S. (2025). Impact of blue economy factors on the sustainable economic growth of China. Environ. Dev. Sustain..

[5] Narwal S., Kaur M., Yadav D.S., Bast F. (2024). Sustainable blue economy: Opportunities and challenges. J. Biosci..

[6] Yan J., Lin J., Yang X., Chen C., Guan X. (2025). Cooperation detection and tracking of underwater target via aerial-surface-underwater vehicles. IEEE Trans. Autom. Control.

[7] Eren F., Pe’eri S., Rzhanov Y., Thein M.W., Celikkol B. (2015). Optical detector array design for navigational feedback between unmanned underwater vehicles (UUVs). IEEE J. Ocean. Eng..

[8] Tang Y., Wang L., Jin S., Zhao J., Huang C., Yu Y. (2023). AUV-based side-scan sonar real-time method for underwater-target detection. J. Mar. Sci. Eng..

[9] Rowley J. Autonomous unmanned surface vehicles (usv): A paradigm shift for harbor security and underwater bathymetric imaging. Proceedings of the OCEANS 2018 MTS/IEEE Charleston.

[10] Wells J.S., Wurth T.J., Manning M.C. Employing a communication payload on an unmanned underwater vehicle (UUV) for harbor monitoring and homeland defense. Proceedings of the Unattended/Unmanned Ground, Ocean, and Air Sensor Technologies and Applications VI.

[11] Conry M., Keefe A., Ober W., Rufo M., Shane D. BIOSwimmer: Enabling technology for port security. Proceedings of the 2013 IEEE International Conference on Technologies for Homeland Security (HST).

[12] Rahman S. (2024). The role of unmanned vehicles in enhancing marine logistics security. Collab. Eng. Dly. Book Ser..

[13] Bakirci M. (2025). Advanced ship detection and ocean monitoring with satellite imagery and deep learning for marine science applications. Reg. Stud. Mar. Sci..

[14] Xu J., Sun X., Zhang D., Fu K. (2014). Automatic detection of inshore ships in high-resolution remote sensing images using robust invariant generalized Hough transform. IEEE Geosci. Remote Sens. Lett..

[15] Jiang L.-B., Wang Z., Hu W.-D. (2011). An AIAC-based inshore ship target detection approach. Remote Sens. Technol. Appl..

[16] Hu J., Xu S., Chen H., Zhang Z. (2009). Detection of ships in harbor in remote sensing image based on local self-similarity. J. Image Graph..

[17] Shi Z., Yu X., Jiang Z., Li B. (2013). Ship detection in high-resolution optical imagery based on anomaly detector and local shape feature. IEEE Trans. Geosci. Remote Sens..

[18] Krizhevsky A., Sutskever I., Hinton G.E. (2017). ImageNet classification with deep convolutional neural networks. Commun. ACM.

[19] Girshick R., Donahue J., Darrell T., Malik J. Rich feature hierarchies for accurate object detection and semantic segmentation. Proceedings of the IEEE Conference on Computer Vision and Pattern Recognition.

[20] Ren S., He K., Girshick R., Sun J. (2016). Faster R-CNN: Towards real-time object detection with region proposal networks. IEEE Trans. Pattern Anal. Mach. Intell..

[21] Liang Y., He R., Li Y., Wang Z. Simultaneous segmentation and classification of breast lesions from ultrasound images using mask R-CNN. Proceedings of the 2019 IEEE International Ultrasonics Symposium (IUS).

[22] Liu Z., Hu J., Weng L., Yang Y. Rotated region based CNN for ship detection. Proceedings of the 2017 IEEE International Conference on Image Processing (ICIP).

[23] Liu W., Anguelov D., Erhan D., Szegedy C., Reed S., Fu C.Y., Berg A.C. Ssd: Single shot multibox detector. Proceedings of the European Conference on Computer Vision.

[24] Wang Y., Wang C., Zhang H. (2018). Combining a single shot multibox detector with transfer learning for ship detection using sentinel-1 SAR images. Remote Sens. Lett..

[25] Redmon J., Divvala S., Girshick R., Farhadi A. You only look once: Unified, real-time object detection. Proceedings of the IEEE Conference on Computer Vision and Pattern Recognition.

[26] Li L., Jiang L., Zhang J., Wang S., Chen F. (2022). A complete YOLO-based ship detection method for thermal infrared remote sensing images under complex backgrounds. Remote Sens..

[27] Hu J., Zhi X., Shi T., Zhang W., Cui Y., Zhao S. (2021). PAG-YOLO: A portable attention-guided YOLO network for small ship detection. Remote Sens..

[28] Chen L., Shi W., Deng D. (2021). Improved YOLOv3 based on attention mechanism for fast and accurate ship detection in optical remote sensing images. Remote Sens..

[29] Li X., Liu L. Patch-Based Coupled Attention Network to Predict MSI Status in Colon Cancer. Proceedings of the International Symposium on Bioinformatics Research and Applications.

[30] Shen A., Zhu Y., Angelov P., Jiang R. (2024). Marine debris detection in satellite surveillance using attention mechanisms. IEEE J. Sel. Top. Appl. Earth Obs. Remote Sens..

[31] Zi N., Li X.M., Gade M., Fu H., Min S. (2024). Ocean eddy detection based on YOLO deep learning algorithm by synthetic aperture radar data. Remote Sens. Environ..

[32] Yang Y., Pan Z., Hu Y., Ding C. (2021). CPS-Det: An anchor-free based rotation detector for ship detection. Remote Sens..

[33] Yao Y., Jiang Z., Zhang H., Zhao D., Cai B. (2017). Ship detection in optical remote sensing images based on deep convolutional neural networks. J. Appl. Remote Sens..

[34] Zhao X., Song Y. (2023). Improved ship detection with YOLOv8 enhanced with MobileViT and GSConv. Electronics.

[35] Gong Y., Chen Z., Deng W., Tan J., Li Y. (2024). Real-time long-distance ship detection architecture based on YOLOv8. IEEE Access.

[36] Pan Z., Li X., Liu L. (2026). A continuously coupled attention neural network for MSI status classification in whole slide images. Biomed. Signal Process. Control.

[37] Huang Y., Han D., Han B., Wu Z. (2025). ADV-YOLO: Improved SAR ship detection model based on YOLOv8. J. Supercomput..

[38] Feng S., Huang Y., Zhang N. (2024). An improved YOLOv8 obb model for ship detection through stable diffusion data augmentation. Sensors.

[39] Bakirci M., Bayraktar I. Assessment of YOLO11 for ship detection in SAR imagery under open ocean and coastal challenges. Proceedings of the 2024 21st International Conference on Electrical Engineering, Computing Science and Automatic Control (CCE).

[40] Li C., Yang Y., Yang X., Chu D., Cao W. (2024). A novel multi-scale feature map fusion for oil spill detection of SAR remote sensing. Remote Sens..

[41] Cheng C., Wang C., Yang D., Wen X., Liu W., Zhang F. (2024). Underwater small target detection based on dynamic convolution and attention mechanism. Front. Mar. Sci..

[42] Prasad D.K., Rajan D., Rachmawati L., Rajabally E., Quek C. (2017). Video processing from electro-optical sensors for object detection and tracking in a maritime environment: A survey. IEEE Trans. Intell. Transp. Syst..

